# Evaluating the Impact of Tobacco Cessation Counseling on Oral Health-Related Quality of Life and Identifying Barriers to Quitting Among Tobacco Smokers

**DOI:** 10.7759/cureus.66072

**Published:** 2024-08-03

**Authors:** Swati Sharma, Siddharth Kapoor, Sahana Shivakumar, Abhishek Mulay, Shivakumar G C, Sameer Kedia

**Affiliations:** 1 Department of Pedodontics and Preventive Dentistry, Rajendra Institute of Medical Sciences (RIMS), Ranchi, IND; 2 Department of Internal Medicine, Rajendra Institute of Medical Sciences (RIMS), Ranchi, IND; 3 Department of Public Health Dentistry, People's College of Dental Sciences and Research Centre, Bhopal, IND; 4 Department of Oral and Maxillofacial Surgery, Yashwantrao Chavan Dental College and Hospital, Ahmednagar, IND; 5 Department of Oral Medicine and Radiology, People’s College of Dental Sciences and Research Centre, Bhopal, IND; 6 Department of Periodontics, Vidarbha Youth Welfare Society's (VYWS) Dental College and Hospital, Amaravati, IND; 7 School of Dentistry, Karnavati University, Gandhinagar, IND

**Keywords:** barriers to quit tobacco, smoking, tobacco cessation, quality of life, oral health

## Abstract

Introduction

Tobacco addiction is widely recognized as the most significant menace to both systemic and oral diseases, resulting in around eight million fatalities worldwide annually. The current investigation was conducted to assess the influence of tobacco cessation counseling on the quality of life linked to oral health and to identify obstacles to quitting among those who use tobacco.

Methods

This observational, follow-up study was carried out among patients referred to the tobacco cessation unit for the cessation of their smoking tobacco habit. Data on the participants was collected in two phases. Oral health-related quality of life (OHRQoL) was assessed at baseline and again three months after quitting smoking habits in the second phase. The assessment of barriers to quitting was done by asking a few questions of all participants. A student t-test and a chi-square test were applied with a p-value <0.05 considering significance.

Results

The study comprised a total of 322 patients, ranging in age from 18 to 62, with a mean age of 32.58 ± 12.901 years. After three months of quitting tobacco, a comparison of the mean scores of the Oral Health Impact Profile (OHIP) domains revealed a significant reduction in the mean score across all domains. The reduction was statistically significant, with a p-value of 0.001. Upon investigating the association between obstacles to quitting tobacco and socioeconomic position, it was discovered that the expense of quitting aids or tobacco programs, as well as the likelihood of weight gain, were strongly linked to the socioeconomic status of the individuals involved in the study.

Conclusion

Based on the results, the present study concluded that oral health-related quality of life significantly improved after quitting.

## Introduction

Tobacco is widely recognized as a significant risk factor for a range of systemic and oral disorders. According to the Global Adult Tobacco Survey (GATS) conducted in 2016-17, the overall prevalence of smoking tobacco use is 10.38% and smokeless tobacco use is 21.38% in India [1]. It has also been stated that tobacco causes death in roughly eight million populations globally per year, making it a huge concern worldwide [2]. Tobacco is used mainly in two forms: smoking tobacco and smokeless tobacco. Smokeless tobacco is a broad word that includes consumption of all kinds, i.e., chewing tobacco, dry snuff, supari, gutkha, zarda, etc. Smokeless tobacco is usually made of a blend of dry tobacco leaves with nicotine, lime, abrasive salts, sweeteners, and more than 4000 compounds. It has been demonstrated that the availability of nicotine is three to four times higher in smokeless tobacco, and chemical utilization in smokeless quid contains more than 30 carcinogens [3,4]. According to the study, the amount of nicotine released in eight-10 chews is similar to 30-40 cigarettes [5]. Smoking tobacco is available in bidis, cigarettes, roll-your-owns, sticks, pipes, water pipes, and kreteks [6]. Various research demonstrated that smoking tobacco is connected with lung cancer, pancreatic cancer, mouth cancer, cardiovascular, and other disorders [7,8]. It has been established that oral and esophageal cancers are listed worldwide as the sixth most prevalent malignancies among both genders; however, as per statistics, oral and esophageal cancers are classified as the third most common type of cancer in nations such as India, Nepal, and the UK [1,9-13]. All forms of tobacco have adverse effects on oral health, including discoloration of teeth, xerostomia, halitosis, erosion of enamel, hypersensitivity, the recession of gingival, poor periodontal health, delayed wound healing, oral mucosal diseases, and carcinomas [13,14].

To combat this, the World Health Organization and the Ministry of Health and Family Welfare created 19 tobacco cessation centers (TCCs) countrywide in 2002 to provide simple access to tobacco cessation services in India [15]. However, the burden of tobacco is dramatically increasing despite these attempts. It has been established that every year a new number of tobacco users is increasing, and according to estimates, every day over 5,500 children start using tobacco and associated products [16]. Through tobacco cessation coaching, dentists can avoid the incidence of different oral and systemic disorders and improve the oral health profile of their patients [17]. Evidence is available showing smoking cessation offers a number of benefits for general health, and stopping smoking improves dental health [18,19].

Oral health-related quality of life is a multidimensional statistic that evaluates not only the absence of disease but also the general well-being associated with oral concerns. It evaluates the effect of oral health on daily activities, psychological well-being, and social interactions. Smokers generally face symptoms such as foul breath, tooth discoloration, and increased plaque buildup, which can lead to more serious oral disorders and negatively influence their self-esteem and social connections. Furthermore, the pain and suffering caused by gingival disease and oral lesions can impede a person's ability to eat and converse, drastically decreasing their quality of life. Any habit is reinforced by many methods; according to Unger et al., good behavior is a complex phenomenon that functions synergistically to adopt good habits and to quit the bad ones [20]. Therefore, the identification of beginning, inciting, and limiting variables in tobacco behaviors is particularly significant. The present study was hence undertaken to evaluate the influence of tobacco cessation counseling on oral health-related quality of life and identify barriers to quitting among smokers.

## Materials and methods

This observational, follow-up study was carried out among patients referred to the tobacco cessation unit of the department (OPD) of Public Health Dentistry, Peoples College of Dental Sciences and Research Centre, Bhopal, India, from June 2023 to December 2023 for the cessation of their tobacco smoking habit. All the protocols of the study were explained to the Ethical Committee of the Institute, and approval was obtained (EC 244255). After explaining the aim and objectives of the study, informed consent was obtained from all eligible participants.

By a convenient sampling technique, patients who registered for a smoking cessation program within the study duration of six months were selected; thus, a total of 350 patients were selected. The study enrolled patients in the cessation clinic within six months, adhering to the following inclusion and exclusion criteria: patients aged over 18 years, habitually smoking tobacco, without systemic diseases or dependence on any systemic medication. We excluded patients with psychiatric medical conditions, as they could potentially impede the counseling process. We excluded patients who were unwilling to participate or not ready to quit. Since this was a follow-up study, some patients did not participate in the second phase of data collection, resulting in a final sample size of 322 patients.

Data collection for this study was conducted in two phases. In the first phase, baseline characteristics were gathered for all participants. Additionally, the frequency of their smoking habits or methods of cessation and their oral health status were assessed. The Oral Health Impact Profile (OHIP-14) questionnaire, which includes 14 questions covering seven domains of oral health, was used for this purpose using a five-point scale [21]. In the second phase, three months after participants had quit smoking, their oral health was reassessed using the OHIP-14 questionnaire. Additionally, barriers to quitting tobacco were evaluated. Participants were asked about various challenges they faced, such as the cost of quitting aid, the expense of cessation programs, concerns about weight gain, difficulty managing stress, peer pressure, increased hunger or thirst, and withdrawal symptoms. All data were collected by a single investigator to reduce observer bias.

Statistical analysis was done using IBM SPSS Statistics for Windows, Version 25 (Released 2017; IBM Corp., Armonk, New York, United States). Data were distributed in frequency distribution tables, and the mean scores of the OHIP-14 domains before and after three months of quitting smoking were calculated by a paired t-test. The association of socioeconomic status with barriers to quitting smoking was calculated by the Chi-square test with a p-value less than 0.05 taken as significant.

## Results

In this study, a total of 322 patients were enrolled within the age group of 18 to 62 years, whose mean age was 32.58 ± 12.901 years. Among 322 patients, 240 (74.5%) were males and 80 (24.8%) were females. When the educational level of patients was assessed, 29 (9%) were illiterate, 132 (41%) completed school, 100 (31.1%) were diploma holders, and 61 (18.9%) had completed academics. As per the socioeconomic status, 11 (3.4%) patients were upper class, 82 (25.5%) were upper-middle class, 84 (26.1%) were lower-middle class, 120 (37.3%) were upper-lower class, and 25 (7.8%) were lower class. A total of 270 (83.9%) participants smoked <20 cigarettes or bidis per day, whereas 52 (16.1%) smoked more than 20 cigarettes or bidis per day. When the method of cessation was assessed, 34 (10.6%) used drugs for cessation, 191 (59.3%) used counseling for cessation, and 97 (30.1%) used combination therapy for cessation (Table 1).

**Table 1:** Demographic details of study participants

Age	Mean age	32.58±12.901
	Min-max	18-62
Gender	Male	242 (74.5%)
	Female	80 (24.8%)
Education level	Illiterate	29 (9%)
	School	132 (41%)
	Diploma	100 (31.1%)
	Academic	61 (18.9%)
Socioeconomic status	Upper	11 (3.4%)
	Upper-middle	82 (25.5%)
	Lower-middle	84 (26.1%)
	Upper-lower	120 (37.3%)
	Lower	25 (7.8%)
Smoking frequency per day	<20	270 (83.9%)
	≥20	52 (16.1%)
Methods of quitting	Drugs	34 (10.6%)
	Counseling	191 (59.3%)
	Combination	97 (30.1%)

When the OHIP-14 questionnaire was assessed, all questions in each domain showed improvement related to the quality of life associated with oral health after three months of quitting smoking (Table 2).

**Table 2:** Frequencies (percentages) of the Oral Health Impact Profile (OHIP-14) questions before and after three months of quitting

Questions	Time interval	Seldom	Sometimes	Fairly often	Very often	All the time	Domains
Q1. Trouble in pronouncing words?	Before	0 (0%)	33 (10.2%)	108 (33.5%)	126 (39.1%)	55 (17.1%)	Functional limitation
	After	108 (33.5%)	118 (36.6%)	51 (15.8%)	27 (8.4%)	18 (5.6%)	
Q2. Worsening of taste	Before	0 (0%)	23 (7.1%)	72 (22.4%)	137 (42.5%)	90 (28%)	
	After	124 (38.5%)	98 (30.4%)	60 (18.6%)	27 (8.4%)	13 (4%)	
Q3. Feeling pain in mouth?	Before	0 (0%)	23 (7.1%)	88 (27.3%)	121 (37.6%)	90 (28%)	Physical pain
	After	108 (33.5%)	122 (37.9%)	47 (14.6%)	27 (8.4%)	18 (5.6%)	
Q4. Discomfort when eating food?	Before	0 (0%)	28 (8.7%)	91 (28.3%)	124 (38.5%)	79 (24.5%)	
	After	108 (33.5%)	109 (33.9%)	60 (18.6%)	27 (8.4%)	18 (5.6%)	
Q5. Unsatisfactory diet?	Before	0 (0%)	51 (15.8%)	123 (38.2%)	99 (30.7%)	49 (15.2%)	Physical disability
	After	0 (0%)	23 (7.1%)	72 (22.4%)	137 (42.5%)	90 (28%)	
Q6. Interrupt meals?	Before	0 (0%)	23 (7.1%)	72 (22.4%)	137 (42.5%)	90 (28%)	
	After	108 (33.5%)	109 (33.9%)	60 (18.6%)	27 (8.4%)	18 (5.6%)	
Q7. Self conscious? Q8. Anxious?	Before	0 (0%)	23 (7.1%)	79 (24.5%)	137 (42.5%)	83 (25.8%)	Psychological discomfort
	After	101 (31.4%)	120 (37.3%)	56 (17.4%)	27 (8.4%)	18 (5.6%)	
	Before	0 (0%)	23 (7.1%)	72 (22.4%)	137 (42.5%)	90 (28%)	
	After	95 (29.5%)	114 (35.4%)	68 (21.1%)	27 (8.4%)	24 (7.5%)	
Q9. Felt uncomfortable?	Before	0 (0%)	23 (7.1%)	72 (22.4%)	137 (42.5%)	90 (28%)	Psychological disability
	After	99 (30.7%)	118 (36.6%)	54 (16.8%)	27 (8.4%)	24 (7.5%)	
Q10. Felt embarrassed?	Before	0 (0%)	28 (8.7%)	80 (24.8%)	135 (41.9%)	79 (24.5%)	
	After	108 (33.5%)	109 (33.9%)	54 (16.8%)	27 (8.4%)	24 (7.5%)	
Q11. Irritable with dealing people?	Before	0 (0%)	23 (7.1%)	72 (22.4%)	137 (42.5%)	90 (28%)	Social disability
	After	101 (31.4%)	116 (36%)	54 (16.8%)	27 (8.4%)	24 (7.5%)	
Q12. Difficulty in doing routine jobs?	Before	0 (0%)	23 (7.1%)	72 (22.4%)	137 (42.5%)	90 (28%)	
	After	101 (31.4%)	116 (36%)	54 (16.8%)	27 (8.4%)	24 (7.5%)	
Q13. Overall less satisfying in life?	Before	0 (0%)	23 (7.1%)	72 (22.4%)	137 (42.5%)	90 (28%)	Handicap
	After	97 (30.1%)	114 (35.4%)	60 (18.6%)	27 (8.4%)	24 (7.5%)	
Q14. Unable to function?	Before	0 (0%)	23 (7.1%)	72 (22.4%)	137 (42.5%)	90 (28%)	
	After	92 (28.6%)	120 (37.3%)	59 (18.3%)	27 (8.4%)	24 (7.5%)	

When a comparison of the mean score of oral health impact profile domains was done before and after three months of quitting smoking, it was found that the mean score was significantly reduced after three months of quitting in all domains with a statistically significant p-value of 0.001. (Table 3)

**Table 3:** Comparison of mean scores before and after three months of quitting tobacco Score range for each domain is 0 - 8; Student t-test was applied and p-value <0.05 was considered significant

	Score before quitting	Score after quitting	Correlation	P-value
Functional limitation	5.54±1.631	2.25±2.223	0.512	0.001
Physical pain	5.65±1.761	2.33±2.286	0.547	0.001
Physical disability	5.55±3.756	4.11±1.808	0.322	0.001
Psychological discomfort	5.78±1.750	2.45±2.245	0.499	0.001
Psychological disability	5.74±1.748	2.48±2.397	0.757	0.001
Social disability	5.83±1.770	2.50±2.393	0.704	0.001
Handicap	5.83±1.771	2.57±2.352	0.721	0.001

Upon evaluating the barriers to tobacco quitting, it was found that 231 (71.7%) patients found that tobacco cessation medication and enrollment in tobacco cessation programs were expensive. Only 18 (5.6%) patients found that tobacco cessation is associated with the risk of gaining weight. A total of 263 (81.7%) patients found that they experienced difficulty handling stress; 254 (78.9%) suffered from peer pressure during the quitting program; and lastly, craving and withdrawal symptoms were associated with 273 (84.8%) patients (Table 4).

**Table 4:** Frequency of barriers to quitting smoking

Questions	Numbers (%)	
	Yes	No
Cost of tobacco cessation aids (medications)	231 (71.7%)	91 (28.3%)
Cost of enrolling in a tobacco cessation program	231 (71.7%)	91 (28.3%)
Risk of putting weight	18 (5.6%)	304 (94.4%)
Difficulty in handling stress	263 (81.7%)	59 (18.3%)
Peer pressure (seeing friends smoking)	254 (78.9%)	68 (21.2%)
Withdrawal symptoms	273 (84.8%)	49 (15.2%)

In the current study, when the association of barriers to tobacco quitting concerning socioeconomic status was assessed, it was found that the cost of quitting aids or tobacco programs and the risk of gaining weight were significantly associated with the socioeconomic status of study participants. While other barriers like stress, peer pressure, craving, and withdrawal symptoms are equally distributed among various socioeconomic statuses (Table 5).

**Table 5:** Association of barriers to smoking quitting with respect to socio-economic status Chi-square test was applied and p-value <0.05 was considered significant

Barriers		Upper (n (%))	Upper-middle (n (%))	Lower-middle (n (%))	Upper-lower (n (%))	Lower (n (%))	P-value
Cost of tobacco cessation aids (medications)	Yes	0 (0%)	2 (2.4%)	84 (100%)	120 (100%)	25 (100%)	0.001
	No	11 (100%)	80 (97.5%)	0 (0%)	0 (0%)	0 (0%)	
Cost of enrolling in a tobacco cessation program	Yes	0 (0%)	2 (2.4%)	84 (100%)	120 (100%)	25 (100%)	0.001
	No	11 (100%)	80 (97.5%)	0 (0%)	0 (0%)	0 (0%)	
Risk of putting weight	Yes	11 (100%)	7 (8.5%)	0 (0%)	0 (0%)	0 (0%)	0.001
	No	0 (0%)	75 (91.4%)	84 (100%)	120 (100%)	25 (100%)	
Difficulty in handling stress	Yes	8 (72.7%)	64 (78.0%)	73 (86.9%)	100 (83.3%)	18 (72%)	0.328
	No	3 (27.2%)	18 (21.9%)	11 (13.0%)	20 (16.6%)	7 (28%)	
Peer pressure (seeing friends smoking)	Yes	8 (72.7%)	63 (76.8%)	65 (77.3%)	100 (83.3%)	18 (72%)	0.608
	No	3 (27.2%)	19 (23.1%)	19 (22.6%)	20 (16.6%)	7 (28%)	
Craving and withdrawal symptoms	Yes	11 (100%)	67 (81.7%)	77 (91.6%)	100 (83.3%)	18 (72%)	0.061
	No	0 (0%)	15 (18.2%)	7 (8.3%)	20 (16.6%)	7 (28%)	

## Discussion

Tobacco use poses significant health risks both systemically and locally, particularly affecting oral health and, consequently, the quality of life-related to oral health (OHRQoL). Smoking tobacco has various harmful effects, including a significantly higher risk of lung cancer, chronic obstructive pulmonary disease (COPD), and heart disease. It also can cause various other cancers, weaken the immune system, and lead to gingival disease, tooth loss, and oral cancer. Smoking can even negatively impact reproductive health, accelerate the aging process, and reduce life expectancy. In total, it severely diminishes quality of life and increases the risk of multiple serious health conditions [21,22]. These conditions can greatly impair functional abilities like chewing and swallowing and affect the cosmetic aspects of dental health, both crucial components of OHRQoL. Given the well-documented effects of tobacco on general and oral health [23,24], this study aimed to evaluate the impact of tobacco cessation counseling on OHRQoL and identify barriers to quitting tobacco. The study included 322 participants aged 18 to 62 years, with an average age of approximately 33 years, enrolled in smoking cessation programs. Among these participants, 240 were men and 80 were women. The cessation methods included medication, counseling, and a combination of both.

The study found that patients' oral health profiles improved across all domains after three months of quitting tobacco. Scores from the OHIP-14 questionnaire indicated enhanced taste, less discomfort when chewing, reduced stress levels, and increased social confidence. These findings align with those of Habibagahi R et al., who also observed improvements in taste, eating, speaking, stress levels, and confidence after quitting tobacco [25]. Other studies have similarly reported that stopping smoking leads to better general and oral health-related quality of life [26,27]. It has been shown that smokers generally have poorer OHRQoL compared to non-smokers [28,29]. According to Souto, M.L.S. et al., smoking cessation therapy is a cost-effective means to improve OHRQoL [30]. In the present study, the most common barrier to quitting smoking was craving and withdrawal symptoms (84.8%), followed by difficulty handling stress (81.7%). When examining the association between socioeconomic status and barriers to quitting, it was found that the cost of enrolling in cessation programs, medications, and the risk of gaining weight were significantly linked to socioeconomic status. Similar findings by Carlson et al. [31] indicated that low socioeconomic status was strongly associated with barriers to quitting, with only the risk of gaining weight being significantly associated with higher-income participants. Other studies have also shown that individuals from low-income groups are more likely to face barriers to quitting tobacco [32,33].

It is well known that smoking can harm almost every organ in the body, lead to numerous diseases, and reduce lifespan while quitting smoking can lower these risks and extend lifespan. In a developing country like India, where resources and health services are limited, "prevention" should be a key strategy to address these issues. Consequently, the Indian government has initiated tobacco cessation programs and established various tobacco cessation centers across the country. Despite these efforts, smoking rates continue to rise, which renders identifying barriers to quitting crucial. This study has certain limitations, such as potential inaccuracies in self-reported data that could introduce biases. Additionally, the study's focus on a specific geographic area may limit the generalizability of the findings, and the follow-up period may not be sufficient to detect long-term health changes. Variations in counseling methodologies could also affect the results.

Dental professionals play a crucial role in identifying people who are addicted to tobacco. Due to their close examination of the oral cavity, they are able to identify early indicators of tobacco use, such as gingival disease, discolored teeth, and lesions suggestive of oral cancer. Dentists can initiate discussions with patients regarding tobacco use and its negative consequences on oral health by creating a safe space in which they feel comfortable disclosing their habits. They can greatly improve their patients' general health and well-being by supporting them in quitting by incorporating tobacco cessation counseling in their routine dental visits. Their early intervention may also prevent tobacco-related oral disorders, which emphasizes their significance in public health initiatives.

## Conclusions

Based on the results, the present study concluded that oral health-related quality of life significantly improved after quitting tobacco. All domains of OHIP, including functional, psychological, and social aspects, were enhanced with reduced pain and discomfort during eating, pronouncing words, stress levels, and social appearance. The cost of enrolling in tobacco cessation programs and their medications, peer pressure, handling stress, and withdrawal symptoms are some important barriers associated with quitting smoking. Socioeconomic status played a significant role in adopting the cost of cessation programs and their medications. We recommended that more research be carried out to identify more precise findings with a larger sample size. As dependency on nicotine has an imperative role in quitting, identification of the role of nicotine is also important.
